# Design of a Practical Metal-Made Cold Isostatic Pressing (CIP) Chamber Using Finite Element Analysis

**DOI:** 10.3390/ma15103621

**Published:** 2022-05-18

**Authors:** Wentao Song, Weicheng Cui

**Affiliations:** 1 Zhejiang University–Westlake University Joint PhD Program, Zhejiang University, Hangzhou 310058, China; songwentao@westlake.edu.cn; 2 Key Laboratory of Coastal Environment and Resources of Zhejiang Province, School of Engineering, Westlake University, Hangzhou 310024, China; 3 Institute of Advanced Technology, Westlake Institute for Advanced Study, Hangzhou 310024, China

**Keywords:** deep-ocean pressure chamber, cold isostatic pressing, pre-stressed wire-wound, finite element analysis, SMART crack growth, unified fatigue life prediction method

## Abstract

The fast development of deep-ocean engineering equipment requires more deep-ocean pressure chambers (DOPCs) with a large inner diameter and ultra-high-pressure (UHP). Using the pre-stressed wire-wound (PSWW) concept, cold isostatic pressing (CIP) chambers have become a new concept of DOPCs, which can provide 100% performance of materials in theory. This paper aims to provide a comprehensive design process for a practical metal-made CIP chamber. First, the generalized design equations are derived by considering the fact that the cylinder and wire have different Young’s moduli and Poisson’s ratios. Second, to verify the theory and the reliability of the CIP chamber, the authors proposed a series of FEA models based on ANSYS Mechanical, including a two-dimensional (2D) model with the thermal strain method (TSM) and a three-dimensional (3D) model with the direct method (DM). The relative errors of the pre-stress coefficient range from 0.17% to 5%. Finally, the crack growth path is predicted by using ANSYS’s Separating Morphing and Adaptive Remeshing Technology (SMART) algorithm, and the fatigue life is evaluated by using the unified fatigue life prediction (UFLP) method developed by the authors’ group. This paper provides a more valuable basis to the design of DOPCs as well as to the similar pressure vessels than the previous work.

## 1. Introduction

Pressure testing with a deep-ocean pressure chamber (DOPC) is a fast and effective method to validate the safety of deep-ocean engineering equipment [1]. To develop full-ocean-depth (FOD) submersibles, large biomimetic robotic fish, new buoyancy materials, and so forth require a DOPC with a large inner diameter and ultra-high-pressure (UHP) [2]. However, such a monobloc chamber must have a large wall thickness. For instance, with a 3 m inner diameter and 90 MPa maximum working pressure (MWP), the *930 Pressure Chamber* of China Ship Scientific Research Center (CSSRC) has a 530 mm wall-thickness, which may reach the ceiling of manufacture [2]. To conduct a FOD level’s pressure testing or tests for buoyancy materials, a DOPC should operate at more than 180 MPa (1.5 times of FOD pressure) or even above 200 MPa. Thus, the structural form of monobloc chambers cannot meet these design requirements of such a DOPC with a large inner diameter [2]. In 2009, the Deep Ocean Exploration and Research (DOER) company developed three innovative DOPCs with the introduced technologies of cold isostatic presses; thus, the DOER company called them cold isostatic pressing (CIP) chambers [3,4]. Using these CIP chambers, DOER conducted a full range of FOD testing for their *Deepsearch* submersible components. The results show that CIP chambers can become a new design concept of DOPCs by replacing the monobloc chambers [2,3].

The limitation of monobloc chambers is that the outer material of cylinders cannot be fully utilized due to the nonuniformity of stress distribution [2]. CIP chambers use the so-called pre-stressed wire-wound (PSWW) concept to establish the compressive stress in the cylinder a priori in order to reduce or even eliminate tensile stress in the cylinder caused by internal working pressures. It can be proved that CIP chambers can provide 100% performance of materials in theory, while monobloc chambers can only utilize 50% of the material’s potential [2]. Furthermore, due to the ingenious structure, the safety of CIP chambers is essentially promoted. From the aspect of the fracture mechanics, even if the fatigue crack nucleates and propagates in the cylinder, it will not extend to the wire-wound layer. Likewise, crack propagation in one wire layer will not affect other layers. Therefore, CIP chambers will only leak before bursting [5,6]. A detailed review of CIP chambers was provided by the authors in Ref. [2].

The American Society of Mechanical Engineers (ASME) Boiler and Pressure Vessel Code (BPVC) provides the linear-elastic equations of the stress distribution of CIP chambers after winding operations, but the design equations to determine the thicknesses of CIP chambers are not provided. Moreover, these equations of the stress distribution are only valid if the Young’s modulus and the Poisson’s ratio of the cylinder and wire are the same, which limits the scope of application [7]. On the one hand, in some industrial fields (e.g., chemical industry, nuclear industry, etc.), the cylinder is usually made of concrete or cemented carbide, for which its Young’s modulus and Poisson’s ratio are quite different from those of the wire-wound layer. On the other hand, although the cylinder and the wire of DOPCs are generally made of high-strength steels, there must be some differences between them in with respect to Young’s modulus (about 5 to 6 GPa) due to the different processes [7,8].

Furthermore, the accurate prediction of the fatigue crack path and estimation of the fatigue life under service loading is very important for the safe and economic design of engineering structures [9]. In this respect, finite element analysis (FEA) has an unquestionable position in modern engineering, which can verify the reliability of structure and provide great insight into various phenomena. The simulation technique involving FEA and the usage of ANSYS Mechanical is an excellent way to save experimental time and cost [10,11]. Recently, engineers have employed the genuine Separating Morphing and Adaptive Remeshing Technology (SMART) algorithm developed by ANSYS to investigate fatigue crack propagation. By using the unstructured mesh method (UMM), a SMART algorithm can automatically update the mesh to reflect changes in crack geometry caused by crack growth at each iteration step, which can drastically reduce the calculation time from a few days to a few minutes [12,13,14,15].

However, there is a lack of research about the FEA model of CIP chambers. In 2014, Wu et al. [16] established an FEA model of a CIP chamber based on ANSYS Mechanical, but this model has inappropriate boundary conditions such that it cannot reflect the effects of PSWW. Wu et al. [16] also evaluated the fatigue life of a CIP chamber by using the stress versus cycles (*S-N*) curve method, but the *S-N* curve method itself is subjected to some theoretical flaws [9]. In 2010, Alegre et al. [17] presented a fatigue analysis of CIP chambers based on the ASME API 579 code for crack assessment and reviewed many different fatigue life prediction methods. Because the scatter of different methods could provide a ten-times difference for practical structures, the currently employed method for evaluation was still not be completely satisfactory [9]. The authors’ group had made some efforts in this area; in 2011, Cui et al. [9] proposed the unified fatigue life prediction (UFLP) method for marine structures, which was validated by the test data of a wide range of metal materials. More detailed information about the UFLP method can be found in Ref. [9].

This paper aims to provide a comprehensive design process for a practical metal-made CIP chamber intended to be constructed in our laboratory at the Deep-Sea Technology Research Center (DSTRC) of Westlake University. First, the generalized design equations of CIP chambers are derived to expand the scope of application, and a case study of the practical CIP chamber is presented. Second, to verify the theory and the reliability of the CIP chamber, the authors proposed a series of FEA models based on ANSYS Mechanical, including a two-dimensional (2D) model with the thermal strain method (TSM) and a three-dimensional (3D) model with the direct method (DM), which has not been previously performed by other researchers. Then, the crack growth path is predicted by using ANSYS’s SMART algorithm, and the fatigue life of the CIP chamber is evaluated by using the UFLP method for the first time. Finally, some profound discussions about design principles of CIP chambers are given, and some conclusions are drawn.

## 2. Theory

### 2.1. Basic Assumptions

**Figure 1:**
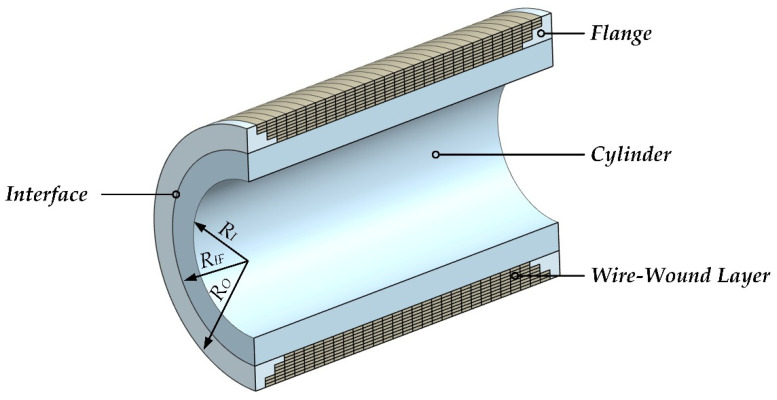
Nomenclature for a CIP chamber: the contact surface between the cylinder and the wire-wound layer is defined as the interface for which its radius is represented as *R_IF_*; *R_I_* and *R_O_* are the inner radius of the cylinder and the outer radius of the CIP chamber, respectively.

Figure 1 shows the nomenclature for a CIP chamber, which consists of a cylinder pre-stressed by a wire-wound layer. The wire-wound layer is formed by a wire helically wounded edge-to-edge in pre-tension many turns around the outside of the cylinder. To deduce the generalized design equations of CIP chambers, the following basic assumptions are introduced.

**Assumption** **1** **(A1).** *The cylinder and the wire are made of two different materials with different Young’s moduli and Poisson’s ratios. Let the Young’s moduli of the cylinder and the wire be E*_1_*and E*_2_*, Poisson’s ratios for them are μ*_1_*and μ*_2_*, and the allowable stresses for them are* [*σ*]_1_ *and* [*σ*]_2_*, respectively*.

**Assumption** **2** **(A2).** *The variation of stresses is under linear-elastic conditions, and yielding is not permitted.*

**Assumption** **3** **(A3).** *The wire is under an ideal winding condition; that is, the wire-wound layer is closely contacted with the cylinder without slipping, and so it is the same with every two adjacent wire layers. Consequently, the displacement continuity condition is satisfied on the interface and the contact surfaces between every two adjacent wire layers.*

According to the above assumptions, the wire-wound layer can be simplified as a cylinder. Thus, the stress distributions of the cylinder and the wire-wound layer can be determined by Lamé formulas, respectively. Lamé formulas represent the stress distribution in a cylinder submitted to uniform internal pressure *P_I_* and external pressure *P_O_*, which takes the following form [18]:

(1) $$\{\begin{array}{c} \sigma_{t}=\frac{R_{I}^{2}}{R_{O}^{2}{-R}_{I}^{2}}\left(1+\frac{R_{O}^{2}}{r^{2}}\right)P_{I}-\frac{R_{O}^{2}}{R_{O}^{2}{-R}_{I}^{2}}\left(1+\frac{R_{I}^{2}}{r^{2}}\right)P_{O}\\ \sigma_{r}=\frac{R_{I}^{2}}{R_{O}^{2}{-R}_{I}^{2}}\left(1-\frac{R_{O}^{2}}{r^{2}}\right)P_{I}-\frac{R_{O}^{2}}{R_{O}^{2}{-R}_{I}^{2}}\left(1-\frac{R_{I}^{2}}{r^{2}}\right)P_{O} \end{array}$$

where *σ_t_* and *σ_r_* are the tangential stress and the radial stress, respectively.

It is easy to understand that the stress state of CIP chambers can be categorized into the pre-stressed state (non-working state, without internal pressure) and the working state [2,19]. In the pre-stressed state, the cylinder will only be under the action of the pre-stress generated by the wire-wound layer, so the stress in this state is known as pre-stress (marked with a superscript “*P*”). If we ignore pre-stress in the entire CIP chamber, the stress generated by the internal pressure can be called the Lamé stress (marked with a superscript “*L*”) because it is determined by Lamé formulas. In the working state, the cylinder will be under the action of both the pre-stress and the internal pressure, so the stress in this state is known as composite stress [2,19].

When designing a CIP chamber, the primary consideration is to what extent we expect to offset the tensile stress in the cylinder. Therefore, we introduce the pre-stress coefficient *η* here [2,19]:

(2) $$\eta =\frac{{|\sigma }_{tI}^{P}|}{\sigma_{tI}^{L}}$$

where $\sigma_{tI}^{P}$ and $\sigma_{tI}^{L}$ are the tangential pre-stress and the tangential Lamé stress on the inner-surface of the cylinder, respectively. Obviously, tensile stress will be eliminated when *η* ≥ 1.

To deduce the generalized design equations of CIP chambers, we let the pre-stress generated by the wire-wound layer to be equivalent to radial stress. Therefore, the cylinder can be regarded as a one that is submitted to uniform pressure on its inner surface and outer surface. In this paper, this equivalent stress is known as interface pressure *P_IF_*.

### 2.2. Generalized Design Equations

#### 2.2.1. Equilibrium Equation

**Figure 2:**
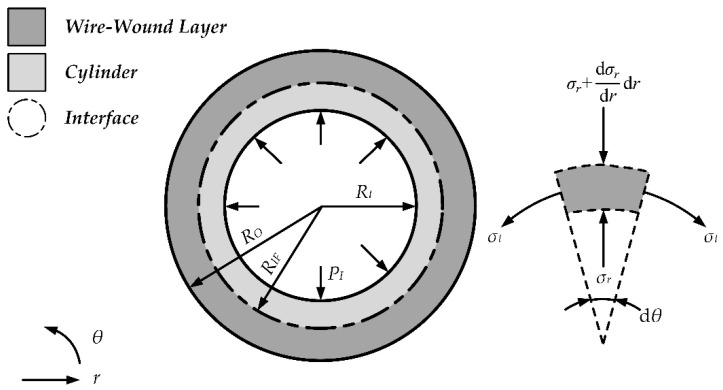
Stress distribution of the wire-wound layer in 2D polar coordinates.

The stress distribution of the wire-wound layer in 2D polar coordinates is depicted in Figure 2, and the equilibrium equation can be expressed as follows.

(3) $$2\left(\sigma_{t}\cdot dr\right)\sin\frac{\theta }{2}+\left(\sigma_{r}+\frac{{d\sigma }_{r}}{dr}\cdot dr\right)\left[2\left(r+dr\right)\sin\frac{\theta }{2}\right]{=\sigma }_{r}\cdot \left(2r\sin\frac{\theta }{2}\right)$$

Then, we have the following.

(4) $$\sigma_{r}=-\frac{1}{r}\int_{r}^{R_{O}}\sigma_{t}\cdot dr$$

The strength criterion of the wire-wound layer has the following form [18,20]:

(5) $$\tau_{max}=\frac{\sigma_{t}{-\sigma }_{r}}{2}\leq \frac{{\left[\sigma \right]}_{2}}{2}$$

where *τ_max_* is the maximum shear stress of the wire-wound layer. The boundary conditions of the wire-wound layer can be described as follows.

(6) $$\{\begin{array}{l} \sigma_{t}\left({r=R}_{O}\right)={\left[\sigma \right]}_{2}\\ \sigma_{r}\left({r=R}_{O}\right)=0 \end{array}$$

Thus, we can obtain the stress distribution of the wire-wound layer in the working state.

(7) $$\{\begin{array}{l} \sigma_{t}={\left[\sigma \right]}_{2}\cdot \left(1+\ln\frac{r}{R_{O}}\right)\\ \sigma_{r}={\left[\sigma \right]}_{2}\cdot \ln\frac{r}{R_{O}} \end{array}$$

When *r* = *R_IF_*, the interface pressure in the working state can be obtained as follows.

(8) $$P_{IF}={\left[\sigma \right]}_{2}\cdot \ln\frac{R_{O}}{R_{IF}}$$

#### 2.2.2. Displacement Continuity Condition

According to basic assumptions, the radial displacement of the outer surface of the cylinder caused by internal pressure *u*_1_ should always be equal to the radial displacement of the inner surface of the wire-wound layer caused by internal pressure *u*_2_. For the cylinder, it is under the action of the internal pressure and the interface pressure. Therefore, according to Lamé formulas, the stress distribution on the interface has the following form:

(9) $$\{\begin{array}{l} \sigma_{tIF}=\frac{{2R}_{I}^{2}}{R_{IF}^{2}{-R}_{I}^{2}}\cdot P_{I}-\frac{R_{IF}^{2}{+R}_{I}^{2}}{R_{IF}^{2}{-R}_{I}^{2}}\cdot P_{IF}\\ \sigma_{rIF}{=-P}_{IF} \end{array}$$

where *σ_tIF_* and *σ_rIF_* are the tangential stress and radial stress on the interface respectively. Thus, according to the elasticity theory [18], we have the following.

(10) $$u_{1}=\frac{R_{IF}}{E_{1}}\left(\sigma_{tIF}{-\mu }_{1}\cdot \sigma_{rIF}\right)=\frac{R_{IF}}{E_{1}}\left(\frac{{2R}_{I}^{2}}{R_{IF}^{2}{-R}_{I}^{2}}\cdot P_{I}-\frac{R_{IF}^{2}{+R}_{I}^{2}}{R_{IF}^{2}{-R}_{I}^{2}}\cdot P_{IF}{+\mu }_{1}\cdot P_{IF}\right)$$

Similarly, for the wire-wound layer, it is only under the action of the interface pressure. Therefore, we have the following.

(11) $$\{\begin{array}{l} \sigma_{tIF}=\frac{R_{O}^{2}{+R}_{IF}^{2}}{R_{O}^{2}{-R}_{IF}^{2}}\cdot P_{IF}\\ \sigma_{rIF}{=-P}_{IF} \end{array}$$

Thus, we can obtain the following.

(12) $$u_{2}=\frac{R_{IF}}{E_{2}}\left(\sigma_{tIF}{-\mu }_{2}\cdot \sigma_{rIF}\right)=\frac{R_{IF}}{E_{2}}\left(\frac{R_{O}^{2}{+R}_{IF}^{2}}{R_{O}^{2}{-R}_{IF}^{2}}\cdot P_{IF}{+\mu }_{2}\cdot P_{IF}\right)$$

As *u*_1_ = *u*_2_, the interface pressure caused by the internal pressure has the following form.

$$P_{IF}=\frac{A_{1}}{A_{2}}\cdot P_{I}$$

(13) $$\{\begin{array}{l} A_{1}=\frac{{2R}_{I}^{2}}{R_{IF}^{2}{-R}_{I}^{2}}\\ A_{2}=\frac{R_{IF}^{2}{+R}_{I}^{2}}{R_{IF}^{2}{-R}_{I}^{2}}+\frac{E_{1}}{E_{2}}\cdot \frac{R_{O}^{2}{+R}_{IF}^{2}}{R_{O}^{2}{-R}_{IF}^{2}}{-\mu }_{1}+\frac{E_{1}}{E_{2}}\cdot \mu_{2} \end{array}$$

#### 2.2.3. Wall-Thickness Equation

According to Lamé formulas, we have the following.

(14) $$\sigma_{tI}^{L}=\frac{R_{IF}^{2}{+R}_{I}^{2}}{R_{IF}^{2}{-R}_{I}^{2}}\cdot P_{I}-\frac{{2R}_{IF}^{2}}{R_{IF}^{2}{-R}_{I}^{2}}\cdot P_{IF}$$

Thus, the strength criterion of the cylinder has the following form.

(15) $$\{\begin{array}{l} \left|\sigma_{tI}^{P}\right|=\eta \cdot \sigma_{tI}^{L}\leq {\left[\sigma \right]}_{1}\\ \sigma_{tI}{=\sigma }_{tI}^{P}{+\sigma }_{tI}^{L}=\frac{1-\eta }{\eta }\cdot \left|\sigma_{tI}^{P}\right|\leq \frac{1-\eta }{\eta }\cdot {\left[\sigma \right]}_{1} \end{array}$$

Replacing Equation (8) with Equations (14) and (15), we can obtain the following.

$$\frac{R_{O}}{R_{IF}}=\exp\left(B_{1}{+B}_{2}\cdot \frac{R_{I}^{2}}{R_{IF}^{2}}\right)$$

(16) $$\{\begin{array}{c} B_{1}=\frac{1}{2{\left[\sigma \right]}_{2}}\left(P_{I}-\frac{1-\eta }{\eta }\cdot {\left[\sigma \right]}_{1}\right)\\ B_{2}=\frac{1}{2{\left[\sigma \right]}_{2}}\left(P_{I}+\frac{1-\eta }{\eta }\cdot {\left[\sigma \right]}_{1}\right) \end{array}$$

Similarly, by replacing Equation (13) with Equations (14) and (15), we can obtain the following.

$$\frac{R_{O}}{R_{IF}}=\sqrt{\frac{C_{1}{+C}_{2}}{C_{1}{-C}_{2}}}$$

(17) $$\{\begin{array}{l} C_{1}=\frac{{4R}_{IF}^{2}\cdot R_{I}^{2}}{{\left(R_{IF}^{2}{-R}_{I}^{2}\right)}^{2}}-\left(\frac{R_{IF}^{2}{+R}_{I}^{2}}{R_{IF}^{2}{-R}_{I}^{2}}{-\mu }_{1}+\frac{E_{1}}{E_{2}}\cdot \mu_{2}\right)\left(\frac{R_{IF}^{2}{+R}_{I}^{2}}{R_{IF}^{2}{-R}_{I}^{2}}-\frac{{\left[\sigma \right]}_{1}}{\eta \cdot P_{I}}\right)\\ C_{2}=\frac{E_{1}}{E_{2}}\left(\frac{R_{IF}^{2}{+R}_{I}^{2}}{R_{IF}^{2}{-R}_{I}^{2}}-\frac{{\left[\sigma \right]}_{1}}{\eta \cdot P_{I}}\right) \end{array}$$

If Equations (16) and (17) are combined, the generalized design equations of CIP chambers take the following form.

(18) $$\frac{B_{1}{+B}_{2}}{B_{1}{-B}_{2}}=\exp\left[2\left(C_{1}{+C}_{2}\cdot \frac{R_{I}^{2}}{R_{IF}^{2}}\right)\right]$$

Thus, interface radius *R_IF_* can be determined by solving Equation (18) with numerical methods, and the outer radius *R_O_* can be determined by substituting *R_IF_* into Equation (16) or Equation (17). Therefore, the wall thickness of the cylinder *δ*_1_ and the thickness of the wire-wound layer *δ*_2_ can be determined, where *δ*_1_ = *R_IF_* − *R_I_* and *δ*_2_ = *R_O_* − *R_IF_*.

#### 2.2.4. Winding Stress Formula

The winding stress of the wire *σ*_0_ is the essential process parameter of CIP chambers, which must be provided in the design. The winding stress is the tangential stress $\sigma_{t}^{P}$ generated in the wire due to pre-tension in the winding process. When the winding process is finished, one wire layer (except the outmost layer) will be under the reaction of the tangential stress $\sigma_{t*}^{P}$ of its adjacent outer layer. Then, we have the following.

(19) $$\sigma_{0}{=\sigma }_{t}^{P}{+\sigma }_{t*}^{P}$$

Moreover, we can obtain the following.

(20) $$\sigma_{t}^{P}{=\sigma }_{t}{-\sigma }_{t}^{L}={\left[\sigma \right]}_{2}\cdot \left(1+\ln\frac{r}{R_{O}}\right)-\frac{R_{IF}^{2}}{R_{O}^{2}{-R}_{IF}^{2}}\left(1+\frac{R_{O}^{2}}{r^{2}}\right)P_{IF}$$

According to Lamé formulas and the displacement continuity condition, we can obtain the reaction tangential stress of the adjacent outer layer as Equation (21). To avoid repetition, the following equations describe the conditions.

$$\sigma_{t*}^{P}=\frac{\left({2D}_{1}{-D}_{2}\right)R_{IF}^{2}{-D}_{2}\cdot r^{2}}{D_{2}\cdot \left(r^{2}{-R}_{IF}^{2}\right)}\left({\left[\sigma \right]}_{2}\cdot \ln\frac{r}{R_{O}}-\frac{R_{IF}^{2}}{R_{O}^{2}{-R}_{IF}^{2}}\cdot \frac{r^{2}{-R}_{O}^{2}}{r^{2}}\cdot \frac{A_{1}}{A_{2}}\cdot P_{I}\right)$$

(21) $$\{\begin{array}{l} D_{1}=\frac{{2r}^{2}}{r^{2}{-R}_{IF}^{2}}\\ D_{2}=\frac{r^{2}{+R}_{IF}^{2}}{r^{2}{-R}_{IF}^{2}}+\frac{E_{2}}{E_{1}}\cdot \frac{R_{IF}^{2}{+R}_{I}^{2}}{R_{IF}^{2}{-R}_{I}^{2}}{+\mu }_{2}-\frac{E_{2}}{E_{1}}\cdot \mu_{1} \end{array}$$

Thus, the winding stress formula takes the following form.

(22) $$\sigma_{0}={\left[\sigma \right]}_{2}\cdot \left[1+2\ln\frac{r}{R_{O}}\cdot \frac{\left(D_{1}{-D}_{2}\right)R_{IF}^{2}}{D_{2}\cdot \left(r^{2}{-R}_{IF}^{2}\right)}\right]-\frac{{2R}_{IF}^{2}\cdot P_{I}}{r^{2}{-R}_{IF}^{2}}\cdot \frac{A_{1}}{A_{2}}\left[1+\frac{D_{1}}{D_{2}}\cdot \frac{\left(r^{2}{-R}_{O}^{2}\right)R_{IF}^{2}}{\left(R_{O}^{2}{-R}_{IF}^{2}\right)r^{2}}\right]$$

#### 2.2.5. Stress Distribution

Based on the above derivation, the generalized stress distribution of CIP chambers has the following form:

1. Cylinder (*R_I_* ≤ *r* ≤ *R_IF_*)

$$\{\begin{array}{c} \begin{array}{c} \sigma_{t}^{P}=-\frac{R_{IF}^{2}}{R_{IF}^{2}{-R}_{I}^{2}}\left(1+\frac{R_{I}^{2}}{r^{2}}\right)\left({\left[\sigma \right]}_{2}\cdot \ln\frac{R_{O}}{R_{IF}}-\frac{A_{1}}{A_{2}}\cdot P_{I}\right)\\ \sigma_{r}^{P}=-\frac{R_{IF}^{2}}{R_{IF}^{2}{-R}_{I}^{2}}\left(1-\frac{R_{I}^{2}}{r^{2}}\right)\left({\left[\sigma \right]}_{2}\cdot \ln\frac{R_{O}}{R_{IF}}-\frac{A_{1}}{A_{2}}\cdot P_{I}\right) \end{array} \end{array}$$

(23) $$\{\begin{array}{c} \sigma_{t}=\frac{R_{I}^{2}}{R_{IF}^{2}{-R}_{I}^{2}}\left(1+\frac{R_{IF}^{2}}{r^{2}}\right)P_{I}-\frac{R_{IF}^{2}}{R_{IF}^{2}{-R}_{I}^{2}}\left(1+\frac{R_{I}^{2}}{r^{2}}\right){\left[\sigma \right]}_{2}\cdot \ln\frac{R_{O}}{R_{IF}}\\ \sigma_{r}=\frac{R_{I}^{2}}{R_{IF}^{2}{-R}_{I}^{2}}\left(1-\frac{R_{IF}^{2}}{r^{2}}\right)P_{I}-\frac{R_{IF}^{2}}{R_{IF}^{2}{-R}_{I}^{2}}\left(1-\frac{R_{I}^{2}}{r^{2}}\right){\left[\sigma \right]}_{2}\cdot \ln\frac{R_{O}}{R_{IF}} \end{array}$$

2. Wire-Wound Layer (*R_IF_* ≤ *r* ≤ *R_O_*)

$$\{\begin{array}{l} \sigma_{t}^{P}={\left[\sigma \right]}_{2}\cdot \left(1+\ln\frac{r}{R_{O}}\right)-\frac{R_{IF}^{2}}{R_{O}^{2}{-R}_{IF}^{2}}\left(1+\frac{R_{O}^{2}}{r^{2}}\right)\frac{A_{1}}{A_{2}}\cdot P_{I}\\ \sigma_{r}^{P}={\left[\sigma \right]}_{2}\cdot \ln\frac{r}{R_{O}}-\frac{R_{IF}^{2}}{R_{O}^{2}{-R}_{IF}^{2}}\left(1-\frac{R_{O}^{2}}{r^{2}}\right)\frac{A_{1}}{A_{2}}\cdot P_{I} \end{array}$$

(24) $$\{\begin{array}{l} \sigma_{t}={\left[\sigma \right]}_{2}\cdot \left(1+\ln\frac{r}{R_{O}}\right)\\ \sigma_{r}={\left[\sigma \right]}_{2}\cdot \ln\frac{r}{R_{O}} \end{array}$$

where $\sigma_{t}^{P}$ and $\sigma_{r}^{P}$ are the tangential pre-stress and radial pre-stress, respectively. According to the elasticity theory [18], the radial displacements of the inner surface of the cylinder in the pre-stressed state and in the working state has the following form.

(25) $$\{\begin{array}{l} u_{I}^{P}=\frac{R_{I}}{E_{1}}\cdot \sigma_{tI}^{P}\\ u_{I}=\frac{R_{I}}{E_{1}}\left(\sigma_{tI}{-\mu }_{1}\cdot \sigma_{rI}\right) \end{array}$$

where $u_{I}^{P}$ and *u_I_* represent the two displacements, respectively. When *E*_1_ = *E*_2_ and *μ*_1_ = *μ*_2_, we can prove that the derived generalized design equations in this paper are equivalent to the equations in the ASME code, which have the following form [7]:

1. Cylinder (*R_I_* ≤ *χ*_1_ ≤ *R_IF_*)

(26) $$\{\begin{array}{c} \sigma_{t}^{P}\left(\chi_{1}\right)=-\left[1+{\left(\frac{R_{I}}{\chi_{1}}\right)}^{2}\right]\int_{R_{IF}}^{R_{O}}\left[\frac{\chi }{\chi^{2}{-R}_{I}^{2}}\cdot \sigma_{0}\left(\chi \right)\right]d\chi \\ \sigma_{r}^{P}\left(\chi_{1}\right)=-\left[1-{\left(\frac{R_{I}}{\chi_{1}}\right)}^{2}\right]\int_{R_{IF}}^{R_{O}}\left[\frac{\chi }{\chi^{2}{-R}_{I}^{2}}\cdot \sigma_{0}\left(\chi \right)\right]d\chi \end{array}$$

2. Wire-Wound Layer (*R_IF_* ≤ *χ*_2_ ≤ *R_O_*)

(27) $$\{\begin{array}{l} \sigma_{t}\left(\chi_{2}\right){=\sigma }_{0}\left(\chi_{2}\right)-\left[1+{\left(\frac{R_{I}}{\chi_{2}}\right)}^{2}\right]\int_{\chi_{2}}^{R_{O}}\left[\frac{\chi }{\chi^{2}{-R}_{I}^{2}}\cdot \sigma_{0}\left(\chi \right)\right]d\chi \\ \sigma_{r}\left(\chi_{2}\right)=-\left[1-{\left(\frac{R_{I}}{\chi_{2}}\right)}^{2}\right]\int_{\chi_{2}}^{R_{O}}\left[\frac{\chi }{\chi^{2}{-R}_{I}^{2}}\cdot \sigma_{0}\left(\chi \right)\right]d\chi \end{array}$$

where *χ* is the radius coordinates, and *χ*_1_ and *χ*_2_ are the radius coordinates of the cylinder and wire-wound layer, respectively.

#### 2.2.6. Pre-Stress Coefficient

The pre-stress coefficient can facilitate the design of CIP chambers, but determining a proper pre-stress coefficient is still a key problem. Because DOPCs are under the action of low-cycle loads, the pre-stress degree of CIP chambers should be essentially dependent upon the requirement of fatigue resistance. As we know, many fatigue tests show that the fatigue resistance of a material increases with increased pre-stress. Moreover, it is further known that fatigue almost never occurs if the stress pulsations are always below the yield stress of a material and stress always remains compressive [5,16]. From the perspective of the fracture mechanics, if the residue stress of the cylinder is always compressive, the stress intensity factor (SIF) must be negative, which can make result in cracks always tending to be closed and restrains the nucleation and propagation of cracks [21]. From the perspective of the elasticity theory, although the tensile stress in the cylinder is eliminated when *η* = 1.0, tensile strain will still exist. Therefore, a proper pre-stress coefficient should eliminate not only the tensile stress but also the tensile strain in the cylinder to reduce the possibility of crack nucleation [2].

According to the elasticity theory [18], we have the following:

(28) $$\{\begin{array}{c} \varepsilon_{tI}^{P}=\frac{1}{E_{1}}\left(\sigma_{tI}^{P}{-\mu }_{1}\cdot \sigma_{rI}^{P}\right)\\ \varepsilon_{tI}^{L}=\frac{1}{E_{1}}\left(\sigma_{tI}^{L}{-\mu }_{1}\cdot \sigma_{rI}^{L}\right) \end{array}$$

where $\varepsilon_{tI}^{P}$ is the tangential strain of the inner-surface of the cylinder in the pre-stressed state, and $\varepsilon_{tI}^{L}$ is the tangential strain of the inner surface of the cylinder generated only by the internal working pressure.

When $\left|\varepsilon_{tI}^{P}\right|$ ≥ $\varepsilon_{tI}^{L}$, there is no tensile strain in the cylinder. Thus, we have the following.

(29) $$\left|\sigma_{tI}^{P}\right|\geq \sigma_{tI}^{L}{+\mu }_{1}\cdot P_{I}=\frac{\left|\sigma_{tI}^{P}\right|}{\eta }{+\mu }_{1}\cdot P_{I}$$

Replacing Equation (15) with Equation (29), the pre-stress coefficient has the following form.

(30) $$\eta =\frac{{\left[\sigma \right]}_{1}}{{\left[\sigma \right]}_{1}{-\mu }_{1}\cdot P_{I}}$$

### 2.3. Case Study

To illustrate how to use the generalized design equations, a case study is presented here. In this case, the design problem is about a CIP chamber with 200 MPa MWP and 500 mm inner diameter intended to be built in our laboratory. According to the ASME code, the design internal pressure *P_I_* should be 1.25 times MWP [7]. The materials of the CIP chamber are all high-strength low alloy steels. Here, the cylinder is forged from the steel *SA-723 Class 2a*, and the wire comprised drawn and cold rolled steel wire *SA-905 Class 2* with a rectangular cross-section of 4.06 mm × 1.02 mm (simplified as 4 mm × 1 mm in design). The materials’ specifications are shown in Table 1 [22]. According to Equation (30), the pre-stress coefficient is 1.074. Therefore, the dimensions of the CIP chamber can be obtained by generalized design equations, which are shown in Table 2. It is known that the cylinder should be wound with 80 layers of wire.

**Figure 3:**
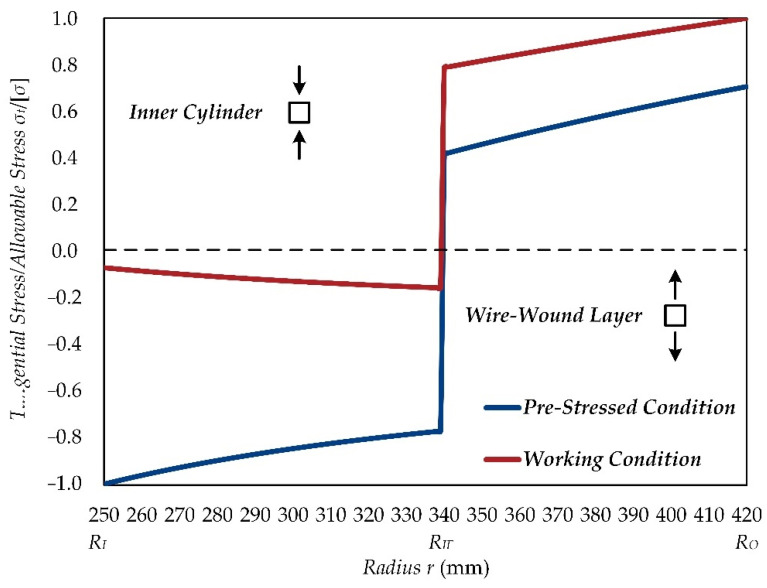
Tangential stress distributions of the CIP chamber.

Figure 3 presents the variation curves of the tangential stress in the CIP chamber, which can provide a better understanding of the design principles and the stress characteristics of CIP chambers. The results are shown as follows:

(1) The tangential stress in the cylinder is compressive when the pre-stress coefficient is greater than 1.0. In the pre-stress state, the tangential stress in the cylinder gradually increases with a decreased radius, and it reaches the maximum on the inner-surface, which is close to the allowable stress of the cylinder. In the working state, the tangential stress caused by the internal pressure is greatly offset and reduced by the residual stress in the cylinder generated due to the pre-stressed wire-wound.
(2) The tangential stress in the wire is always tensile stress. In the pre-stress state, tangential stress gradually increases with an increased radius of the wire. In the working state, the tangential stress further increases due to internal pressure, and reaches the maximum on the outermost layer, which is close to the allowable stress of the wire.
(3) The tangential stress on the inner surface of the cylinder shall not exceed its allowable stress, and this principle can guarantee full use of the cylinder’s material. Likewise, the tangential stress in the outermost wire layer shall not exceed its allowable stress, and this principle can guarantee full use of the wire’s material.

**Figure 4:**
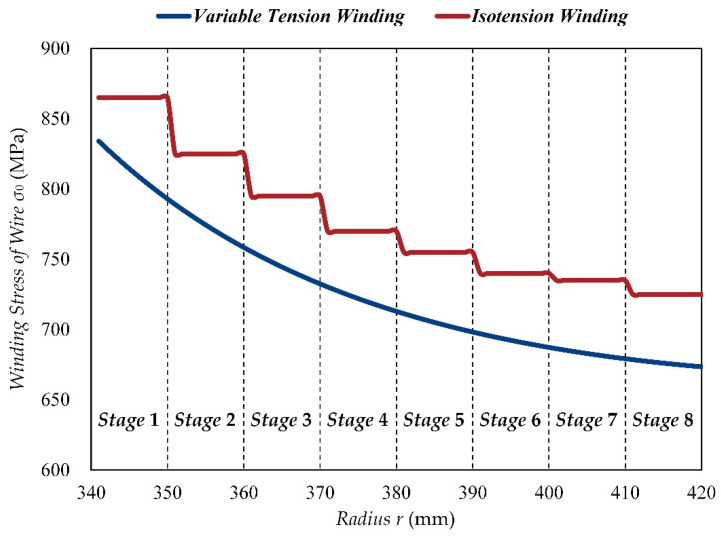
The variation curves of the winding stress of the CIP chamber.

The winding stress of the wire is a function of radius *r*, which can be determined by Equation (22). However, the variable tension control in the winding process is very difficult to achieve. In order to reduce the difficulty and the cost of the winding process, the eighty layers of the wire are simplified as eight isotension stages, and each wire layer in one isotension stage is wound with equal tension instead of variable tension [16]. The actual values of the winding stress in each isotension stage should be appropriately increased based on the theoretical value of the central layer of the stage to compensate for the loss of pre-stress coefficient caused by isotension winding, which are shown in Table 3 and Figure 4.

## 3. Finite Element Analysis

### 3.1. Finite Element Model

To verify the theory and reliability of the CIP chamber in the above case, the authors proposed a series of FEA models based on ANSYS Mechanical. The key point of FEA modeling is how to impose the winding stress as the boundary condition to simulate the effect of PSWW. Therefore, TSM is applied to the 2D model, and the DM proposed by the authors is used in the 3D model.

The TSM originated from a proposed methodology of wire winding simulation conducted by Alegre et al. [23]. The basic idea of this methodology is to convert the elastic strains of each wire layer of a CIP chamber into the corresponding thermal strains, which take the following form [23]:

(31) $$\varepsilon_{T}\approx -\frac{\sigma_{0}}{E_{2}}\left(1+\frac{\delta_{w}}{r}\cdot \frac{r^{2}{+R}_{I}^{2}}{r^{2}{\;-\;R}_{I}^{2}}\right)$$

where *ε_T_* is the equivalent thermal strain, and *δ_w_* is the thickness of the wire.

According to the theory of ANSYS [24], the thermal strain can be expressed as follows:

(32) $$\varepsilon_{T}{=\alpha }_{T}\cdot \left({T\;-\;T}_{0}\right)$$

where *α_T_* is the material’s isotropic secant coefficient of thermal expansion (in 1/°C), *T*_0_ is the initial temperature (in °C), and *T* is the thermal load (in °C). By combining Equation (31) with (32), the equivalent thermal loads can be converted from the winding stresses of the wire, which are shown in Table 4.

**Figure 5:**
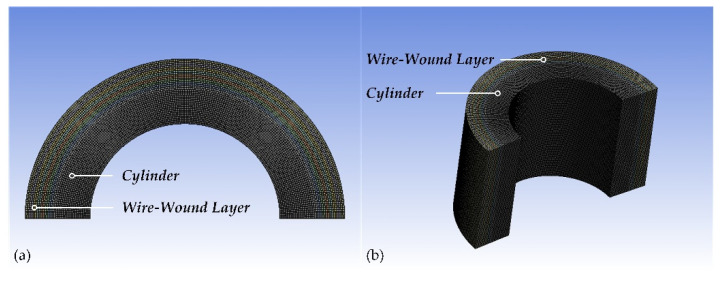
The FEA 2D model of the CIP chamber with 5 mm element size: (**a**) plane strain (1/2 graphical expansion) and (**b**) axisymmetric (1/2 graphical expansion with 500 mm length).

In the FEA 2D model, the wire-wound layer of CIP chambers is simplified into eight iso-tension stages. According to the stress characteristics of CIP chambers, the finite element types are set to be plane strain and axisymmetric, respectively, shown in Figure 5. The element size is optimized by using a mesh sensitivity analysis and the quadratic elements with 5 mm are used to improve the precision of the results. The frictional type of contacts is used, and the frictional coefficient is 0.1. In this setting, the two contacting geometries can carry shear stresses up to a certain magnitude across their contact face before they start sliding relative to each other [24]. The Augmented Lagrange formulation is used for contact pairs to provide a better performance, which takes the following form [24]:

(33) $$F_{n}=\{\begin{array}{ll} K_{n}\cdot u_{n}{+\lambda }_{i+1} & \mathrm{if}\;u_{n}\leq 0\\ 0 & \mathrm{if}\;u_{n}>0 \end{array}$$

where *F_n_* is the normal contact force, *K_n_* is the contact normal stiffness, *u_n_* is the contact gap size, and *λ_i_*_+1_ is the Lagrange multiplier force at iteration *i* + 1.

The boundary conditions of the wire are imposed by the thermal loads in Table 4. Then, the related results can be obtained by using the post-processing function of ANSYS Mechanical, which will be provided in the next subsection. The main parameters of the ANSYS Mechanical are listed in Table 5.

**Figure 6:**
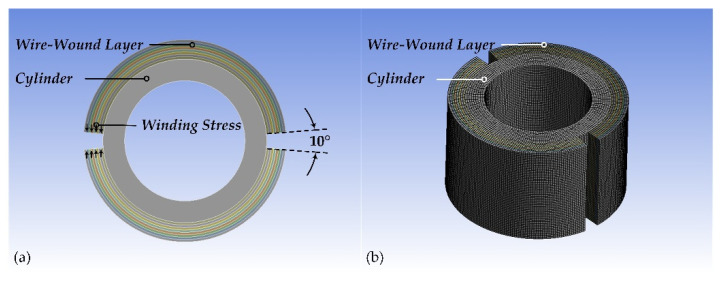
The FEA 3D model of the CIP chamber with 500 mm length and 10 mm element size: (**a**) geometric model and (**b**) meshed model.

Using TSM, the proposed 2D model can reflect the stress changes of the cylinder, but it cannot present the stress distribution of the wire-wound layer itself. Obviously, the wire winding of CIP chambers is a typical 3D problem. Therefore, a 3D model is also proposed by the authors. To impose the winding stress of the wire, the wire-wound layer is divided into two symmetric parts with a notch of 10°. Thus, the 3D model can directly impose the winding stress of the wire as the boundary condition, and this is the meaning of the direct method, shown in Figure 6. The element size is also optimized by using a mesh sensitivity analysis and the quadratic elements with 10 mm are used to improve the precision of the results. The main parameters of the ANSYS Mechanical are very similar to those of the 2D model (also see Table 5), and the related results will also be provided in the next subsection.

### 3.2. Results

**Figure 7:**
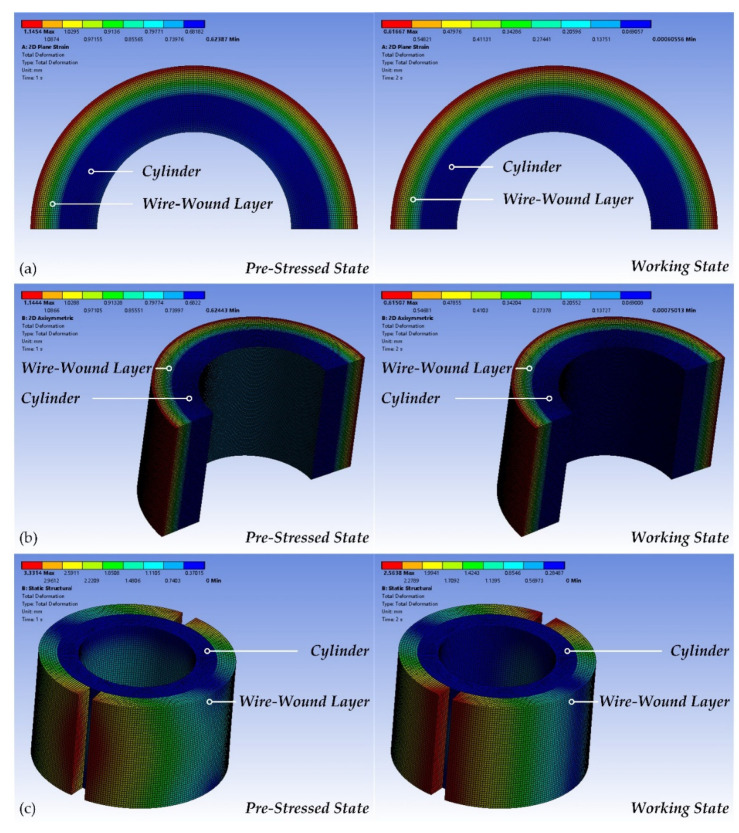
The deformation distributions of the CIP chamber: (**a**) 2D plane strain model with 1/2 graphical expansion, (**b**) 2D axisymmetric model with 1/2 graphical expansion and 500 mm length), and (**c**) 3D model.

Table 6 provides the main results obtained by the above proposed FEA models, and Figure 7 presents the deformation distributions of the CIP chamber. The results are shown as follows:

(1) The deformation distributions of the CIP chamber obtained by ANSYS all present in a rainbow image, and the maximum displacement of the CIP chamber appears on the outermost wire layer, which is in accordance with the theory. In the FEA 2D model, the displacement of the inner surface of the cylinder in the pre-stressed state is about 0.68 mm, while in the working state, it decreased to about 0.048 mm. In the FEA 3D model, the displacement of the inner surface of the cylinder in the pre-stressed state is about 0.73 mm, while in the working state it decreased to about 0.049 mm. The deformation results obtained by the FEA models are very close to the theory values, and the maximum relative errors are about 4.3% in the 2D model and 2.9% in the 3D model.
(2) The FEA 2D model can only present the stress distribution of the cylinder. In the pre-stressed state, the maximum stress of the cylinder is the tangential stress on the inner-surface of the cylinder, which is about −585 MPa with a relative error of 5%. In the working state, the tangential stress on the inner-surface of the cylinder decreased to about −41 MPa with a maximum relative error of 8.6%. The pre-stress coefficient is about 1.13 with a relative error of 5%.
(3) The FEA 3D model can present stress distributions of both the cylinder and the wire-wound layer. In the pre-stressed state, the maximum stress of the wire-wound layer is the tangential stress in the outermost wire layer, which is about 678.64 MPa with a relative error of only 0.78%. In the working state, this maximum stress increased to about 832 MPa with a relative error of 12.7%. The pre-stress coefficient is about 1.076 with a relative error of only 0.17%.
(4) Two FEA models can also provide interface pressure. In the 2D model, the maximum relative errors range from 0.3% to 3.2%, while the maximum relative errors range from 3.2% to 8.6% in the 3D model.

Therefore, the proposed FEA models of the CIP chamber can reflect the effect of PSWW, and these results confirm the correctness of the generalized design equations derived in this paper. Overall, the FEA 2D model using TSM has a fast calculation speed (several minutes) with slightly larger errors, while the FEA 3D model using DM has higher accuracy with a very slow calculation speed (tens of hours). However, the FEA 3D model can directly impose the winding stress as the boundary condition and can present the stress distributions not only in the cylinder but also in the wire-wound layer, which is a more realistic simulation model.

### 3.3. Fatigue Crack Propagation

As the above-mentioned discussion, the inner cylinder of the CIP chamber can be considered to have infinite fatigue life when the pre-stress coefficient is greater than 1.0. Thus, the fatigue strength of the CIP chamber mainly depends on the steel wire, which is always under the action of low-cyclic tensile stress. The stress ratio *R* of the steel wire can be defined by *σ_min_*/*σ_max_*, where *σ_min_* and *σ_max_* are the minimum stress level and the maximum stress level, respectively. In the above case, the stress ratio of the steel wire is about 0.7.

**Figure 8:**
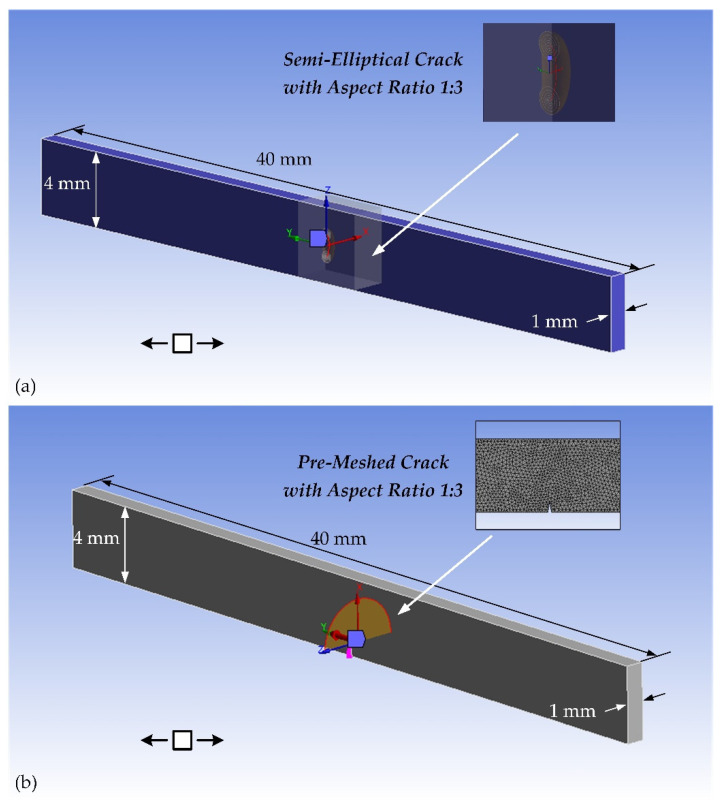
The FEA model of SMART crack growth of a piece of the steel wire used in the CIP chamber with (**a**) a semi-elliptical crack and (**b**) a V-notch pre-meshed crack. The steel wire is under the tensile cycle load. And here, the *x*-axis points to the direction of the crack extension, the *y*-axis points to the normal direction of the crack surface, and the *z*-axis direction is the tangential direction of the crack surface.

To illustrate how to predict the crack growth path of the steel wire and to evaluate the fatigue life of the CIP chamber, a FEA model of SMART crack growth of a piece of the steel wire used in the CIP chamber is proposed based on ANSYS Mechanical, which is shown in Figure 8. In Case 1, the initial crack is set to be a semi-elliptical crack with the initial depth of 0.2 mm and the aspect ratio of 1:3 on the side of the steel wire [17]. In Case 2, the initial crack is set to be a V-notch pre-meshed crack with the initial depth of 0.2 mm and the aspect ratio of 1:3 at the bottom of the steel wire [17]. In ANSYS’s SMART algorithm, the fatigue life is evaluated by using the Paris equation, which takes the following form [15,21]:

(34) $$\frac{da}{dN}=C\cdot {\left(\Delta K\right)}^{m}$$

where *a* is the crack length, *N* is the load cycles, d*a*/d*N* represents the crack growth rate, Δ*K* is the stress intensity factor (SIF) range, *C* and *m* are experimentally determined constants. Here, *C* = 2.29 × 10^−10^ m/cycle (MPa·m^1/2^)^−*m*^ and *m* = 2 [15].

**Figure 9:**
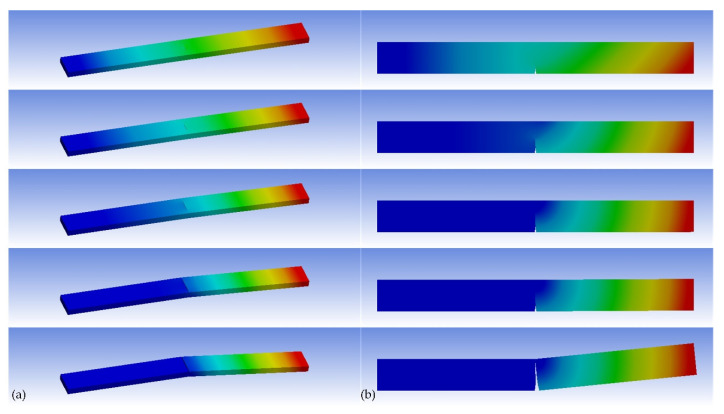
The predicted crack growth trajectories of the steel wire used in the CIP chamber: (**a**) a semi-elliptical crack (60 substeps) and (**b**) a V-notch pre-meshed crack (20 substeps).

The stress level of the outermost layer in the CIP chamber (here is 953 MPa) is imposed in the FEA model. The predicted trajectories of the crack growth can be obtained by increasing the number of substeps in ANSYS Mechanical (see Figure 9). The results are shown as follows:

(1) The semi-elliptical crack on the side of the steel wire will gradually be opened and extended under the cyclic tensile load. The steel wire will be fractured along the direction of its thickness, and bending failure will eventually occur.
(2) The V-notch at the bottom of the steel wire will also gradually be opened and extended under the cyclic load. The steel wire will be fractured along the direction of its height, and shear failure will eventually occur.
(3) ANSYS’s SMART algorithm can provide a very fast computing method as well as a better visualization for fatigue crack growth. The calculation time of Case 1 is only about 3.5 h under the condition of 60 substeps while that of Case 2 is only ten minutes under the condition of 20 substeps.
(4) The predicted fatigue life of the steel wire with the semi-elliptical crack is about 147,696 while that of the steel wire with the V-notch is about 17,007. This suggests that the fatigue life of the steel wire will be greatly affected by the crack at the bottom of the steel wire. Therefore, in the total productive maintenance (TPM) of the CIP chamber, the non-destructive testing (NDT) of the crack at the bottom of the steel wire should be paid more attention to.

### 3.4. Unified Fatigue Life Prediction Method

To provide an accurate prediction of the fatigue life of the CIP chamber, the UFLP method is applied here. The general constitutive relation in the UFLP method takes the following form [9]:

$$\frac{da}{dN}=\frac{A{\left[K_{max}\cdot \left({1-f}_{op}\right){-\Delta K}_{effth}\right]}^{m}}{1-{\left(\frac{K_{max}}{K_{C}}\right)}^{n}}$$

(35) $$\{\begin{array}{l} K_{max}=\sqrt{{\pi r}_{e}\left[\sec\left(\frac{\pi }{2}\cdot \frac{\sigma_{max}}{\sigma_{v}}\right)+1\;\right]}\cdot \left[1+Y\left(a\right)\sqrt{\frac{a}{{2r}_{e}}}\right]\cdot \sigma_{max}\\ K_{min}=\sqrt{{\pi r}_{e}\left[\sec\left(\frac{\pi }{2}\cdot \frac{\sigma_{max}}{\sigma_{v}}\right)+1\;\right]}\cdot \left[1+Y\left(a\right)\sqrt{\frac{a}{{2r}_{e}}}\right]\cdot \sigma_{min}\\ {\Delta K=K}_{max}{-K}_{min} \end{array}$$

where *K_max_* and *K_min_* are the maximum SIF level and the minimum SIF level, respectively, *K_C_* is the actual fracture toughness, Δ*K_effth_* is the threshold effective SIF range, *f_op_* is the crack opening function, *A* is a material and environmentally sensitive constant of dimensions in the crack growth rate model, *m* is a material constant, and *n* is an index indicating the unstable fracture. *r_e_* is an empirical material constant of the inherent flaw length, *σ_v_* is the virtual strength representing the material strength at the condition of *r_e_* = 0, and *Y*(*a*) is the geometrical factor.

The actual fracture toughness *K_C_* can be determined by the following equation [9]:

(36) $$K_{C}=\left[\frac{{\left(1-2\mu \right)}^{2}-\sqrt{{1-\mu }^{2}}}{{\left(1-2\mu \right)}^{2}-1}\cdot \frac{\pi \lambda }{{\left(1-2\mu \right)}^{2}}+\frac{\sqrt{{1-\mu }^{2}}-1}{{\left(1-2\mu \right)}^{2}-1}\right]\cdot K_{IC}$$

where *K_IC_* is the plane strain fracture toughness, and *λ* is the crack tip plastic zone confident. The value of *λ* can be calculated by the follwing equation [9]:

(37) $$\{\begin{array}{l} \lambda =F+\frac{\frac{1}{\pi }-\frac{1}{2.2p}{\left(\frac{1}{\pi }\right)}^{\frac{1}{p}}-F}{{\left[1+\frac{\delta_{w}\cdot \sigma_{Y}^{2}}{K_{max}^{2}}\cdot \frac{p}{1+p}\right]}^{1.6+\frac{1}{p}}}\\ F=\frac{{\left(1-1.65\mu \right)}^{2}}{5}-\frac{1}{20p}{\left[{\left(1-1.65\mu \right)}^{2}\right]}^{\frac{1}{p}} \end{array}$$

where *p* is the strain hardening exponent of the material. The value of *K_IC_* can be estimated by the following equation [9]:

(38) $$K_{IC}=\left(16.348-0.0685\cdot \varepsilon_{f}\right)\cdot {\Delta K}_{th0}$$

Where Δ*K_th_*_0_ is the threshold SIF range under zero load ratio, and *ε_f_* is the fracture strain of material. When 0.5 ≤ *R* < 1, the value of Δ*K_th_*_0_ can be determined by the following equation [9]:

(39) $${\Delta K}_{th0}=\frac{{\Delta K}_{th}}{{\left(1.05-1.4R+0{.6R}^{2}\right)}^{0.3}}$$

where Δ*K_th_* is the threshold SIF range. It should be observed that *K_max_*, *K_C_*, and *f_op_* are all the functions of crack length *a*.

**Figure 10:**
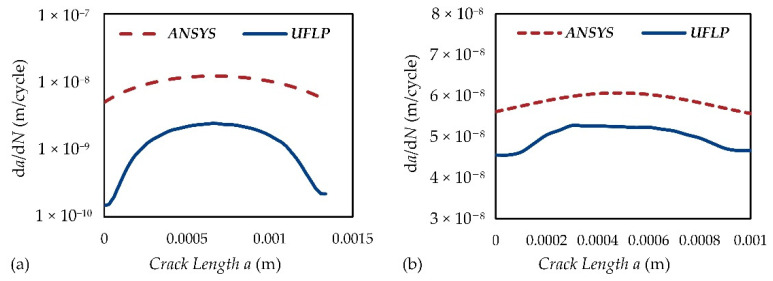
The comparison curves of the crack growth rate obtained by (**a**) ANSYS and (**b**) the UFLP method.

Based on the results of FEA, the fatigue life of the CIP chamber can be predicted more accurately by using the UFLP method. The related parameters are estimated according to Ref. [9] and the ASME code [7,8,22,25], which are shown in Table 7. The comparison curves of the crack growth rate obtained by ANSYS and the UFLP method are shown in Figure 10, and the predicted fatigue life of the CIP chamber is provided in Table 8. The results show that the predicted fatigue life can be significantly different by using different methods. Since ANSYS’s SMART algorithm is only based on the Paris equation, the direct results of ANSYS Mechanical are more conservative. However, we can still use the values of SIF obtained in ANSYS Mechanical to provide a more convincing result by applying the UFLP method. Anyhow, the shear failure of the steel wire is always the most dangerous situation.

## 4. Discussion

The fast development of deep-ocean technologies needs more DOPCs with a large inner diameter and UHP. As the working pressure and the inner diameter increases, the wall-thickness of monobloc chambers will be increased dramatically. The fundamental defect of monobloc chambers is that the outer material of chambers cannot be fully utilized. With the PSWW concept, CIP chambers are widely regarded to be the most reliable and durable pressure chambers ever designed [2]. Moreover, when a fatigue crack nucleates in CIP chambers, the consequences of fracture would not be as sudden and violent as it would be in the case for monobloc chambers. This is because the fatigue cracks in the cylinder will not continue to propagate through the wire-wound layer, and correspondingly, a fatigue crack at one point of the wire will not immediately initiate new cracks [5,6].

The stress distribution equations of CIP chambers in the ASME code limit the scope of application. Therefore, the generalized design equations were derived considering that the cylinder and the wire have different Young’s moduli and Poisson’s ratios. To reduce the difficulty and the cost of the winding process, numerous wire layers should be simplified as a few isotension stages, and the isotension winding is used in each wire layer instead of variable tension winding [16]. However, the winding stress should be appropriately increased to compensate the loss of the pre-stress coefficient caused by isotension winding.

The critical stress of a CIP chamber should be analyzed by FEA in the design stage. The fatigue life of CIP chambers mainly depends on the steel wire if the design pre-stress coefficient is greater than 1.0. The UFLP method proposed by Cui et al. can explain most of the observed macroscopic fatigue phenomena satisfactorily, and it can provide an effective method for evaluating the fatigue life of CIP chambers. As a matter of fact, more than 90% of over 1900 deployed CIP chambers since the 1960s can still operate well today [2]. Thus, the fatigue life of CIP chambers must not be estimated conservatively due to their ingenious structure. In other words, a CIP chamber behaves as though it was manufactured from a stronger material than it actually was. In addition, the shear failure of the steel wire should always be paid more attention to.

## 5. Conclusions

**Figure 11:**
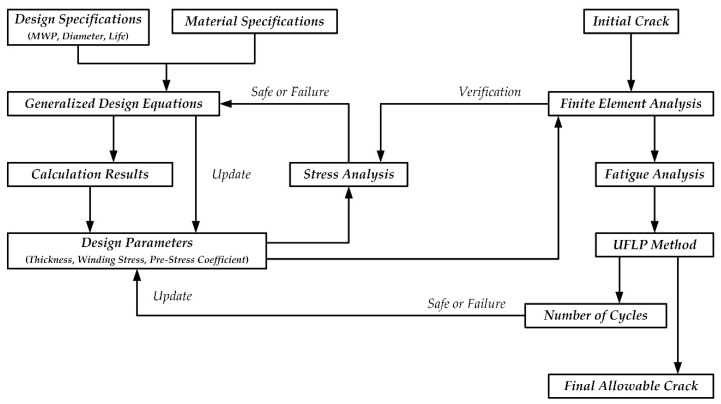
Schematic illustration of the design procedure of CIP chambers.

A comprehensive design process of a practical metal-made CIP chamber intended to be constructed in our laboratory was presented in this paper, which can be illustrated in Figure 11. The generalized design equations of CIP chambers were derived by the authors. To illustrate how to use the design equations, a case study of the practical CIP chamber was given, and the stress distribution of the CIP chamber was investigated. To verify the theory and the reliability of the CIP chamber, the authors proposed a series of FEA models based on ANSYS Mechanical, including a 2D model using TSM and a 3D model using DM. Furthermore, ANSYS’s SMART algorithm was used to investigate two different types of cracks, including a semi-elliptical crack and a V-notch pre-meshed crack. To predict the fatigue life of the CIP chamber accurately, the UFLP method is applied instead of the very simplified Paris equation. This paper can provide more valuable basis to the design of DOPCs in marine engineering as well as to similar pressure vessels than the previous work.

The main conclusions can be summarized as follows:

(1) The generalized design equations of CIP chambers are derived by considering the fact that the cylinder and wire have different Young’s moduli and Poisson’s ratios, which expands the scope of application.
(2) To increase the fatigue resistance of CIP chambers, the design pre-stress coefficient should be slightly greater than 1.0, which can guarantee that the residual stress of the cylinder is always compressive. The tangential stress on the inner surface of the cylinder shall not exceed its allowable stress, which can guarantee full use of the cylinder’s material. Likewise, the tangential stress in the outermost wire layer shall not exceed its allowable stress, which can guarantee full use of the wire’s material.
(3) The proposed FEA models of the CIP chamber can reflect the effect of PSWW, including a 2D model with TSM and a 3D model with DM. The 2D model has a fast calculation speed with slightly larger errors, while the 3D model has higher accuracy with a very slow calculation speed. However, the 3D model can present stress distributions not only in the cylinder but also in the wire-wound layer, which is a more realistic simulation model.
(4) The deformation results obtained by the FEA models are very close to the theory values, and the maximum relative errors are about 4.3% in the 2D model and 2.9% in the 3D model. The stress results obtained by the FEA model are also very close to the theory values, and the maximum relative errors of the pre-stress coefficient are about 5% in the 2D model and 0.17% in the 3D model. These results confirm the correctness of the generalized design equations derived in this paper.
(5) ANSYS’s SMART algorithm can provide an inspiring method as well as a better visualization to predict fatigue crack growth. The calculation time of the semi-elliptical crack is only about 3.5 h under the condition of 60 substeps while that of the V-notch pre-meshed crack is only ten minutes under the condition of 20 substeps.
(6) The predicted fatigue life can be significantly different by using different methods. The fatigue life of the CIP chamber predicted by the UFLP method is about 12 times that predicted by the Paris equation in maximum. According to engineering practices, the UFLP method is well suited for evaluating the fatigue life of CIP chambers while the direct results of ANSYS Mechanical are more conservative.

## Figures and Tables

**Table 1:** Material specifications of the high strength low alloy steels used in the CIP chamber [22].

Materials	Young’s Modulus *E*	Poisson’s Ratio *μ*	Tensile Stress *σ_T_*	Yield Strength *σ_Y_*	Allowable Stress [*σ*] ^1^
SA-723 Class 2a	195 GPa	0.3	1000 MPa	895 MPa	559 MPa
SA-905 Class 2	201 GPa	0.3	1770 MPa	1525 MPa	953 MPa

^1^ The allowable stress is determined by *σ_Y_*/*n_s_*, where *n_s_* is the safety factor (here, *n_s_* = 1.6) [2,19].

**Table 2:** Design parameters of the CIP chamber.

Dimensions	Numerical Results	Design Values ^1^
Wall-Thickness of The Cylinder *δ*_1_	104 mm	90 mm
Thickness of The Wire-Wound Layer *δ*_2_	85.5 mm	80 mm
Total Thickness of The CIP Chamber *δ*	190 mm	170 mm
Outer Diameter of The Cylinder *D_IF_*	708 mm	680 mm
Outer Diameter of The CIP Chamber *D_O_*	879 mm	840 mm

^1^ The design values must ensure that the maximum stress in the CIP chamber does not exceed the allowable stress.

**Table 3:** Winding stresses of the wire in the CIP chamber.

Order Number of Stages	Stage 1	Stage 2	Stage 3	Stage 4	Stage 5	Stage 6	Stage 7	Stage 8
Number of Wire Layers	1~10	11~20	21~30	31~40	41~50	51~60	61~70	71~80
Theoretical Values (MPa) ^1^	814.43	774.45	744.52	721.99	705.02	692.3	682.88	676.04
Actual Values (MPa)	865	825	795	770	755	740	735	725

^1^ Determined by Equation (22) at the central position of each isotension stage in the radial direction.

**Table 4:** The equivalent thermal loads used in the FEA 2D model of the CIP chamber.

Order Number of Stages	Stage 1	Stage 2	Stage 3	Stage 4	Stage 5	Stage 6	Stage 7	Stage 8
Winging Stress (MPa)	865	825	795	770	755	740	735	725
Equivalent Thermal Load (°C)	43.41	41.37	39.84	38.56	37.79	37.03	36.76	36.25

**Table 5:** The main parameters of ANSYS Mechanical in two FEA models of the CIP chamber.

FEA Model	2D Model	3D Model
Element Type	*PLANE183*	*SOLID186*
Element Order	Quadratic	Quadratic
Element Size	5 mm	10 mm
Contact Type	Frictional	Frictional
Contact Formulation	Augmented Lagrange	Augmented Lagrange
Number of Elements	3574 (plane strain) 3400 (axisymmetric)	183,416
Number of Nodes	12,901 (plane strain) 12,077 (axisymmetric)	1,051,920
Number of Steps	2	2
Boundary Conditions	Step 1: only thermal loads Step 2: thermal loads and internal pressure	Step 1: only winding stresses Step 2: winding stresses and internal pressure

**Table 6:** Main results of the CIP chamber obtained by performing theoretical calculations and FEA models.

Physical Quantities	Theory	2D Plane Strain		2D Axisymmetric		3D Model	
		ANSYS	Error	ANSYS	Error	ANSYS	Error
$\sigma_{tI}^{P}$	−556.68 MPa	−585.23 MPa	5.13%	−585.66 MPa	5.21%	−557.63 MPa	0.17%
$\sigma_{rI}^{P}$	0 MPa	−0.05 MPa	-	0.06 MPa	-	−0.32 MPa	-
$\sigma_{tO}^{P}$	673.42 MPa	-	-	-	-	678.64 MPa	0.78%
$\sigma_{rO}^{P}$	0 MPa	-	-	-	-	0.04 MPa	-
$\sigma_{tI}$	−38.3 MPa	−40.91 MPa	6.82%	−41.59 MPa	8.61%	−39.68 MPa	3.62%
$\sigma_{rI}$	−250 MPa	−249.98 MPa	0.01%	−249.98 MPa	0.01%	−250.05 MPa	0.02%
$\sigma_{tO}$	953 MPa	-	-	-	-	832.05 MPa	12.69%
$\sigma_{rO}$	0 MPa	-	-	-	-	−0.09 MPa	-
*P_IF_*_1_ ^1^	201.38 MPa	207.74 MPa	3.16%	207.87 MPa	3.22%	218.71 MPa	8.61%
*P_IF_*_2_ ^2^	73.52 MPa	73.29 MPa	0.32%	73.33 MPa	0.26%	75.86 MPa	3.18%
$u_{I}^{P}$	0.7137 mm	0.6828 mm	4.33%	0.6833 mm	4.25%	0.7341 mm	2.85%
$u_{I}$	0.0471 mm	0.0481 mm	2.18%	0.0473 mm	0.49%	0.0485 mm	2.89%
*η*	1.0739	1.1289	5.13%	1.1298	5.21%	1.0757	0.17%

^1^ *P_IF_*_1_ represents the interface pressure in the working state. ^2^ *P_IF_*_2_ represents the interface pressure caused only by internal working pressure.

**Table 7:** The parameters used in the UFLP method to predict the fatigue life of the CIP chamber [7,8,22,25].

*A*	*m*	*n*	*p*	Δ*K_th_*	Δ*K*_*th*0_	*K_IC_*	*ε_f_*
1.39 × 10^−10^	2.3668	6	0.0598	2.7955 MPa	3.7972 MPa	62.0634 MPa	0.05

**Table 8:** The predicted fatigue life of the CIP chamber.

Crack Case	Paris Equation	UFLP Method
Semi-Elliptical Crack	147,696 cycles	1,807,125 cycles
V-Notch	17,007 cycles	20,026 cycles

## Data Availability

Not applicable.

## Nomenclature

*A*	A material and environmentally sensitive constant of dimensions in the crack growth rate model
*A*_1_	A constant to calculate the interface pressure
*A*_2_	A constant to calculate the interface pressure
*B*_1_	A constant in the wall-thickness equations
*B*_2_	A constant in the wall-thickness equations
*C*	A material constant [m/cycle (MPa·m^1/2^)^−*m*^]
*C*_1_	A constant in the wall-thickness equations
*C*_2_	A constant in the wall-thickness equations
*D*_1_	A constant to calculate the winding stress of the wire
*D*_2_	A constant to calculate the winding stress of the wire
*E*_1_	The Young’s modulus of the cylinder (MPa)
*E*_2_	The Young’s modulus of the wire (MPa)
*F_n_*	Normal contact force (N)
*K_C_*	Actual fracture toughness (MPa·m^1/2^)
*K_IC_*	Plane strain fracture toughness (MPa·m^1/2^)
*K_max_*	Maximum stress intensity factor (MPa·m^1/2^)
*K_min_*	Minimum stress intensity factor (MPa·m^1/2^)
*K_n_*	Contact normal stiffness (N/mm)
*N*	Load cycles (cycle)
*P_I_*	Internal pressure (MPa)
*P_IF_*	Interface pressure (MPa)
*P*_*IF*1_	The interface pressure in the working state (MPa)
*P*_*IF*2_	The interface pressure caused only by internal working pressure (MPa)
*P_O_*	External pressure (MPa)
*R*	Stress ratio
*R_I_*	The inner radius of the cylinder (mm)
*R_IF_*	The radius of the interface (mm)
*R_O_*	The outer radius of the CIP chamber (mm)
*T*	Thermal load (°C)
*T*_0_	Initial temperature (°C)
*a*	Crack length (m)
d*a*/d*N*	Crack growth rate (m/cycle)
*f_op_*	A crack opening function
*m*	A constant representing the slope of the corresponding fatigue crack growth rate curve in the crack growth rate model
*n*	An index indicating the unstable fracture in the crack growth rate model
*p*	The strain hardening exponent of the material
*r*	Polar radius in polar coordinates
*r_e_*	An empirical material constant of the inherent flaw length (m)
*u*_1_	The radial displacement of the outer-surface of the cylinder caused by the internal pressure (mm)
*u*_2_	The radial displacement of the inner-surface of the wire-wound layer caused by the internal pressure (mm)
*u_I_*	The radial displacements of the inner-surface of the cylinder in the working state (mm)
$u_{I}^{P}$	The radial displacements of the inner-surface of the cylinder in the pre-stess state (mm)
*u_n_*	Contact gap size (mm)
Δ*K*	Stress intensity factor range (MPa·m^1/2^)
Δ*K_effth_*	Threshold effective stress intensity factor range (MPa·m^1/2^)
Δ*K_th_*	Threshold stress intensity factor range (MPa·m^1/2^)
Δ*K_th_*_0_	The Threshold stress intensity factor range under zero load ratio (MPa·m^1/2^)
*α_T_*	Isotropic secant coefficient of thermal expansion (1/°C)
*δ*_1_	The wall-thickness of the cylinder (mm)
*δ*_2_	The wall-thickness of the wire-wound layer (mm)
*δ_w_*	The thickness of the wire (mm)
*ε_T_*	Equivalent thermal strain (mm/mm)
*ε_f_*	Fracture strain of materials (m/m)
$\varepsilon_{tI}^{L}$	The tangential strain of the inner-surface of cylinder generated only by the internal working pressure (mm/mm)
$\varepsilon_{tI}^{P}$	The tangential strain of the inner-surface of the cylinder in the pre-stressed state (mm/mm)
*η*	Pre-stress coefficient
*θ*	Polar angle in polar coordinates
*λ*	Crack tip plastic zone coefficient
*λ_i_*_+1_	The Lagrange multiplier force at iteration *i* + 1 (N)
*μ*_1_	The Poisson’s ratio of the cylinder
*μ*_2_	The Poisson’s ratio of the wire
*σ*_0_	The winding stress of the wire (MPa)
*σ_max_*	Maximum stress level (MPa)
*σ_min_*	Minimum stress level (MPa)
*σ_r_*	Radial stress (MPa)
$\sigma_{r}^{P}$	Radial pre-stress (MPa)
*σ_rIF_*	The radial stress on the interface (MPa)
*σ_t_*	Tangential stress (MPa)
*σ_tIF_*	The tangential stress on the interface (MPa)
$\sigma_{t}^{L}$	Tangential Lamé stress (MPa)
$\sigma_{tI}^{L}$	The tangential Lamé stress on the inner-surface of the cylinder (MPa)
$\sigma_{t}^{P}$	Tangential pre-stress (MPa)
$\sigma_{t*}^{P}$	Reaction tangential pre-stress (MPa)
$\sigma_{tI}^{P}$	The tangential pre-stress on the inner-surface of the cylinder (MPa)
*σ_v_*	The virtual strength of the material representing the material strength at the condition of *r_e_* = 0 (MPa)
[*σ*_1_]	The allowable stress of the cylinder (MPa)
[*σ*_2_]	The allowable stress of the wire (MPa)
*τ_max_*	Maximum shear stress (MPa)
*Y*(*a*)	A geometrical factor to calculate the stress intensity factors
*χ*	The radius coordinates in the ASME equations
*χ*_1_	The radius coordinates of the cylinder in the ASME equations
*χ*_2_	The radius coordinates of the wire-wound layer in the ASME equations
